# Gene Expression Analyses during Spontaneous Reversal of Cardiomyopathy in Mice with Repressed Nuclear CUG-BP, Elav-Like Family (CELF) Activity in Heart Muscle

**DOI:** 10.1371/journal.pone.0124462

**Published:** 2015-04-20

**Authors:** Twishasri Dasgupta, Ryan J. Coram, Samantha J. Stillwagon, Andrea N. Ladd

**Affiliations:** Department of Cellular and Molecular Medicine, Lerner Research Institute, Cleveland Clinic, Cleveland, Ohio, United States of America; University of Valencia, SPAIN

## Abstract

CUG-BP, Elav-like family (CELF) proteins regulate cell type- and developmental stage-specific alternative splicing in the heart. Repression of CELF-mediated splicing activity via expression of a nuclear dominant negative CELF protein in heart muscle was previously shown to induce dysregulation of alternative splicing, cardiac dysfunction, cardiac hypertrophy, and dilated cardiomyopathy in MHC-CELFΔ transgenic mice. A “mild” line of MHC-CELFΔ mice that expresses a lower level of the dominant negative protein exhibits cardiac dysfunction and myopathy at a young age, but spontaneously recovers normal cardiac function and heart size with age despite the persistence of splicing defects. To the best of our knowledge, this was the first example of a genetically induced cardiomyopathy that spontaneously recovers without intervention. In this study, we explored the basis for this recovery. We examined whether a transcriptional program regulated by serum response factor (SRF) that is dysregulated in juvenile MHC-CELFΔ mice is restored in the mild line with age, and evaluated global changes in gene expression by microarray analyses. We found that differences in gene expression between the mild line and wild type hearts are greatly reduced in older animals, including a partial recovery of SRF target gene expression. We did not find evidence of a new compensatory pathway being activated in the mild line with age, and propose that recovery may occur due to developmental stage-specific compatibility of CELF-dependent splice variants with the cellular environment of the cardiomyocyte.

## Introduction

CUG-BP, Elav-like family (CELF) proteins regulate cell type- and developmental stage-specific alternative splicing in the heart [1]. Repression of CELF function or over-expression of CELF proteins in heart muscle leads to cardiomyopathy in transgenic mice [2,3,4], and dysregulation of CELF-mediated alternative splicing has been implicated in human cardiac pathogenesis in myotonic dystrophy and diabetic cardiomyopathy [5,6,7]. In addition to regulating cardiac gene expression directly through the alternative splicing of cardiac transcripts, we recently demonstrated that perturbing CELF activity in the hearts of transgenic mice also affects cardiac gene expression indirectly via modulation of the transcriptional activity of serum response factor (SRF) [8]. Repression of nuclear CELF activity in heart muscle reduced expression of homeodomain only protein X (HOPX) and four and a half LIM domain-containing protein 2 (FHL2), proteins which bind to SRF and inhibit its activity [9,10,11], and increased the expression of SRF target genes. Conversely, over-expression of CELF1 induced FHL2 expression and reduced the expression of SRF target genes. SRF is a key cardiac transcription factor involved in regulating heart development, aging, contractile apparatus structure and function, and cardiac hypertrophy [12,13,14,15]. Thus, CELF proteins represent an important regulatory node for controlling cardiac gene expression at transcriptional and post-transcriptional levels during normal development and disease.

In MHC-CELFΔ transgenic mice, a nuclear dominant negative CELF protein (NLSCELFΔ) is expressed under control of the mouse α-myosin heavy chain promoter, which drives specific expression in postnatal heart muscle [4]. CELF-mediated alternative splicing is specifically dysregulated in MHC-CELFΔ hearts, and this dysregulation can be rescued by over-expression of the cardiac CELF protein, CELF1 [4]. Two lines of MHC-CELFΔ mice, the MHC-CELFΔ-10 (“severe”) line and MHC-CELFΔ-574 (“mild”) line, express different amounts of NLSCELFΔ protein and exhibit different degrees of cardiac pathogenesis that correlate with the extent to which the splicing of CELF targets is dysregulated [4,16]. Both lines develop cardiac hypertrophy, dilated cardiomyopathy, and impaired cardiac function, all of which are greater in the severe line. In addition, myocytolysis, fibrosis, and elevated mortality rates are observed in the severe, but not mild, line. CELF proteins are thought to drive some fetal-to-adult transitions in alternative splicing [17,18], and the onset of cardiomyopathy in MHC-CELFΔ mice is consistent with an important role in early postnatal remodeling in the heart [16]. Longitudinal analyses of MHC-CELFΔ mice also revealed a surprising recovery of cardiac function, dilation, and hypertrophy in the mild line over time without a loss of NLSCELFΔ expression, change in endogenous CELF protein expression, or reversal of splicing defects [16]. The heart does not improve with age in the severe line, probably because early muscle loss and fibrosis is too extensive to recover. To the best of our knowledge, the recovery of the mild line of MHC-CELFΔ mice was the first example of a genetically induced cardiomyopathy that spontaneously recovered without intervention. The basis for this recovery, however, has remained a mystery.

In this study, we examined whether a normalization of SRF activity could contribute to recovery in the MHC-CELFΔ mild line. We found that the SRF inhibitors HOPX and FHL2 were expressed at higher levels in the mild line at 24 weeks than at 3 weeks, but remained lower than wild type. Some, but not all, SRF target genes returned to normal levels at 24 weeks, indicating that there is a partial restoration of the SRF pathway in the mild line with age. Global differences in gene expression were also compared between wild type and MHC-CELFΔ mice at 3 weeks and 24 weeks by microarray. Consistent with the reversal of cardiomyopathy in the mild line, many genes that were dysregulated at 3 weeks returned to normal levels at 24 weeks. In contrast, global gene expression in the severe line (which does not recover) became more disrupted with age. Only a handful of changes occurred in the mild line specifically at 24 weeks, and these are not predicted to be beneficial. Thus, there is no evidence of a new compensatory pathway being activated in the mild line. This supports a model in which developmental stage-specific compatibility of CELF-dependent splice variants dictates their effects on cardiac health and function.

## Materials and Methods

### Ethics statement

This study was conducted under the approval of the Cleveland Clinic Institutional Animal Care and Use Committee (Protocol number 2011–0493) and in strict accordance with the recommendations of the American Veterinary Medical Association. Mice were euthanized by carbon dioxide inhalation followed by cervical dislocation to ensure death, consistent with the guidelines of the American Veterinary Medical Association Panel on Euthanasia. All efforts were made to minimize pain and distress during animal husbandry and euthanasia.

### Transgenic mice

MHC-CELFΔ-10 (“severe”) and MHC-CELFΔ-574 (“mild”) transgenic lines were maintained as hemizygotes. Wild type littermates were used for sex- and age-matched controls. Genotyping was performed by PCR as previously described [4]. Because males exhibit a lower penetrance of the phenotype than females in both lines [4], only females were used in this study.

### Western blotting

Western blots were performed as previously described [8]. All western blots shown are representative of three independent sets. Equivalent loading was confirmed both by stripping and reprobing for GAPDH, and by Ponceau S staining of the membrane. Quantification was performed using ImageQuant TL software. Pairwise comparisons of means from the three sets were performed via one-tailed t-tests assuming unequal variances using Microsoft Excel software. Differences were considered statistically significant when P ≤ 0.05.

### Real-time RT-PCR

Real-time RT-PCR experiments were performed as previously described [8]. Primers sequences are provided in S1 Table. All real-time RT-PCR reactions were performed in triplicate on samples from at least three different individuals per group. Data was analyzed with StepOne software v2.1 using the comparative CT method. Fold changes are reported as the mean + standard error of the mean. Statistical comparisons of means were performed via one-tailed t-tests assuming unequal variances using Microsoft Excel software. Differences were considered statistically significant when P ≤ 0.05.

### Microarray analysis

Whole hearts were excised postmortem from 3 or 24 week-old female mice, and RNA samples were prepared as previously described [8]. Two wild type mice from each line were used at both 3 and 24 week time points (n = 4 total), three each of MHC-CELFΔ-10 and MHC-CELFΔ-574 transgenic mice were used at 3 weeks, and five each of MHC-CELFΔ-10 and MHC-CELFΔ-574 transgenic mice were used at 24 weeks. cDNA library preparation and hybridization to MouseWG-6 v2.0 Expression BeadChips (Illumina) were performed by the Cleveland Clinic Lerner Research Institute’s Genomics Core. The microarray data for this study have been deposited in NCBI’s Gene Expression Omnibus [19], and are accessible through GEO Series accession number GSE62363 (http://www.ncbi.nlm.nih.gov/geo/query/acc.cgi?acc=GSE62363). The new microarray data were combined with our previously generated data set (GEO accession number GSE40677; [8]) from 3 week-old wild type (n = 6) and transgenic mice (n = 3 per line) in order to increase the sample size for greater statistical power. Statistical comparisons of the data, pathway and gene ontology analyses were performed by the Cleveland Clinic Bioinformatics Core as previously described [8]. An FDR p-value threshold of < 0.05 or raw p-value of < 0.001 and absolute fold change threshold of 2.0 were used to identify significantly affected genes.

## Results and Discussion

### SRF-dependent gene expression partially recovers in the mild line at 24 weeks

**Fig 1:**
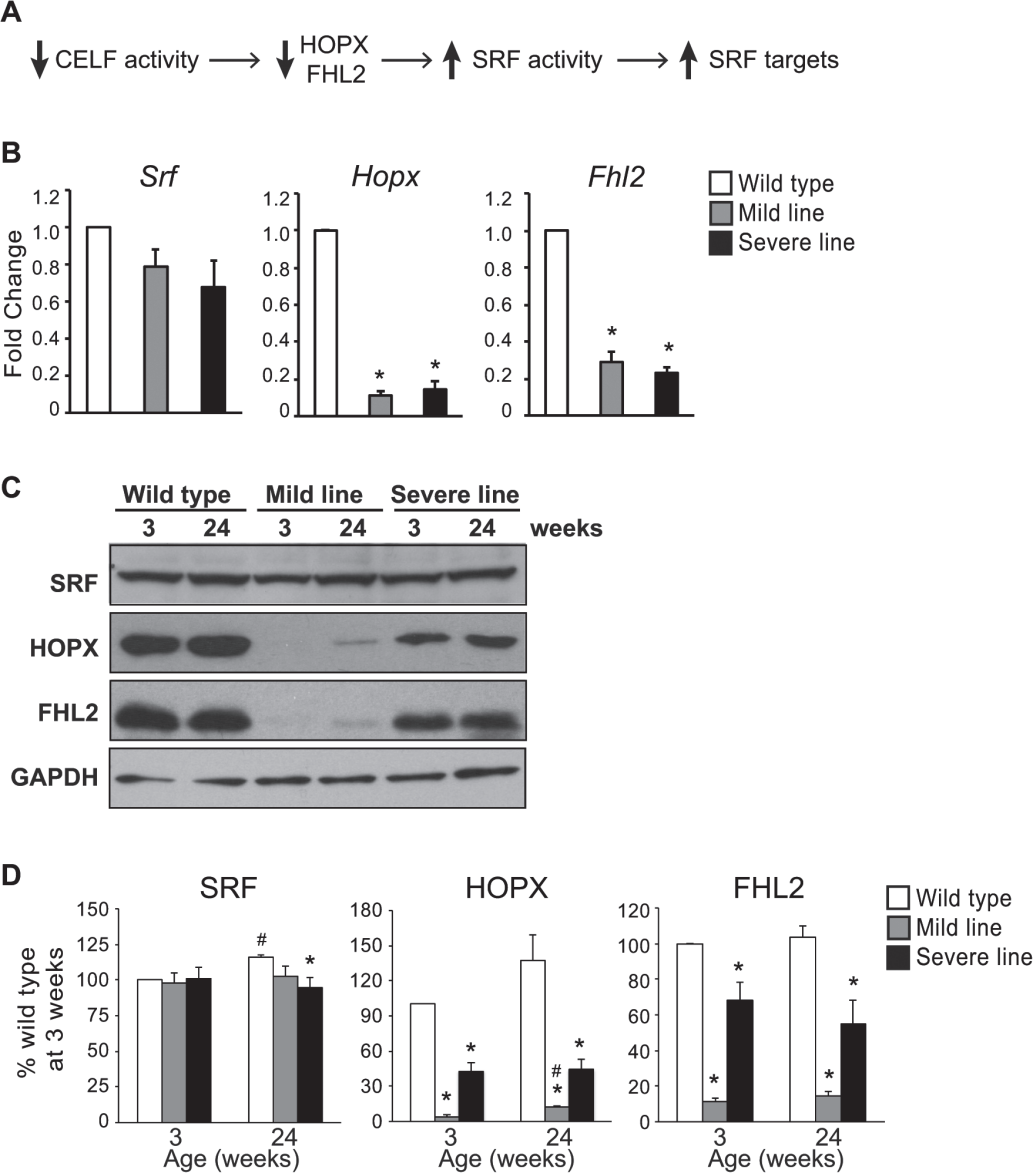
*Hopx* and *Fhl2* remain down-regulated in MHC-CELFΔ mice at 24 weeks. (A) We previously demonstrated that *Hopx* and *Fhl2* transcripts and proteins are down-regulated in MHC-CELFΔ mice at 3 weeks [8]. HOPX and FHL2 are known inhibitors of the cardiac transcription factor SRF, and reductions in HOPX and FHL2 are accompanied by increases in SRF target genes without a corresponding increase in SRF levels. (B) Total RNA was extracted from the hearts of 24 week-old wild type and MHC-CELFΔ mice, and mRNA levels were assayed by qRT-PCR using SYBR green-based detection. While *Srf* transcripts were not significantly changed, *Hopx* and *Fhl2* remain down-regulated in the hearts of both lines of MHC-CELFΔ mice at 24 weeks. Fold changes shown represent the mean + standard error of the mean of three independent sample sets. An asterisk indicates a significant difference from wild type (P ≤ 0.05). (C) Representative western blots of wild type, MHC-CELFΔ mild and severe line females at 3 and 24 weeks of age. Equivalent loading was further confirmed by Ponceau S staining (data not shown). (D) Quantitation of western blots (n = 3) shows an increase in HOPX expression in transgenic mice from the mild line at 24 weeks compared to 3 weeks, although levels still remain reduced compared to wild type hearts at the equivalent age. There is a trend towards increased FHL2 levels in the mild line at 24 weeks as well, but it is not statistically significant compared to the mild line at 3 weeks, and remains significantly lower than wild type. HOPX and FHL2 levels do not change over time in the severe line, and remain lower than wild type. SRF levels vary only slightly, and do not differ between wild type and the mild line at 3 or 24 weeks. An asterisk indicates a significant difference from wild type mice at the same age, and a pound sign indicates a significant difference from mice of the same group at 3 weeks (P ≤ 0.05).

We previously demonstrated that *Hopx* and *Fhl2* transcripts and their corresponding proteins are reduced in MHC-CELFΔ mice compared to wild type littermates at 3 weeks [8]. This reduction in HOPX and FHL2 is accompanied by an increase in the expression of several SRF target genes despite no difference in SRF levels, presumably due to the alleviated inhibition of SRF activity (Fig 1A). We proposed that the combined effects of dysregulating the SRF transcriptional program and dysregulating the alternative splicing of CELF targets both contribute to cardiac dysfunction in MHC-CELFΔ mice [8]. In the mild line (MHC-CELFΔ-574), the state of the heart improves with age such that by 24 weeks the ejection fraction, fractional shortening, chamber dilation, wall thickness, and heart size are all comparable to wild type [16]. This recovery does not occur because splicing is restored to normal, however, as CELF targets continue to exhibit aberrant splicing patterns at 24 weeks [16]. To test whether normalization of the SRF program could be contributing to recovery in the mild line, we evaluated *Srf*, *Hopx*, and *Fhl2* expression in transgenic females at 24 weeks of age (Fig 1B). As seen at 3 weeks, *Hopx* and *Fhl2* transcripts are still significantly reduced in both mild and severe lines compared to wild type, while *Srf* remains at normal levels. SRF, HOPX, and FHL2 protein levels were also compared between transgenic and wild type animals by western blot (Fig 1C and 1D). As previously reported [8], HOPX and FHL2 levels were strongly reduced in MHC-CELFΔ mice at 3 weeks. Interestingly, HOPX levels were higher in transgenic hearts of the mild line at 24 weeks than at 3 weeks, although they remained lower than wild type. FHL2 levels also showed a trend towards increased levels at 24 weeks compared to 3 weeks, though this difference was not statistically significant. In contrast, HOPX and FHL2 levels did not change with age in either the wild type or severe line mice, where levels were higher to start with.

**Fig 2:**
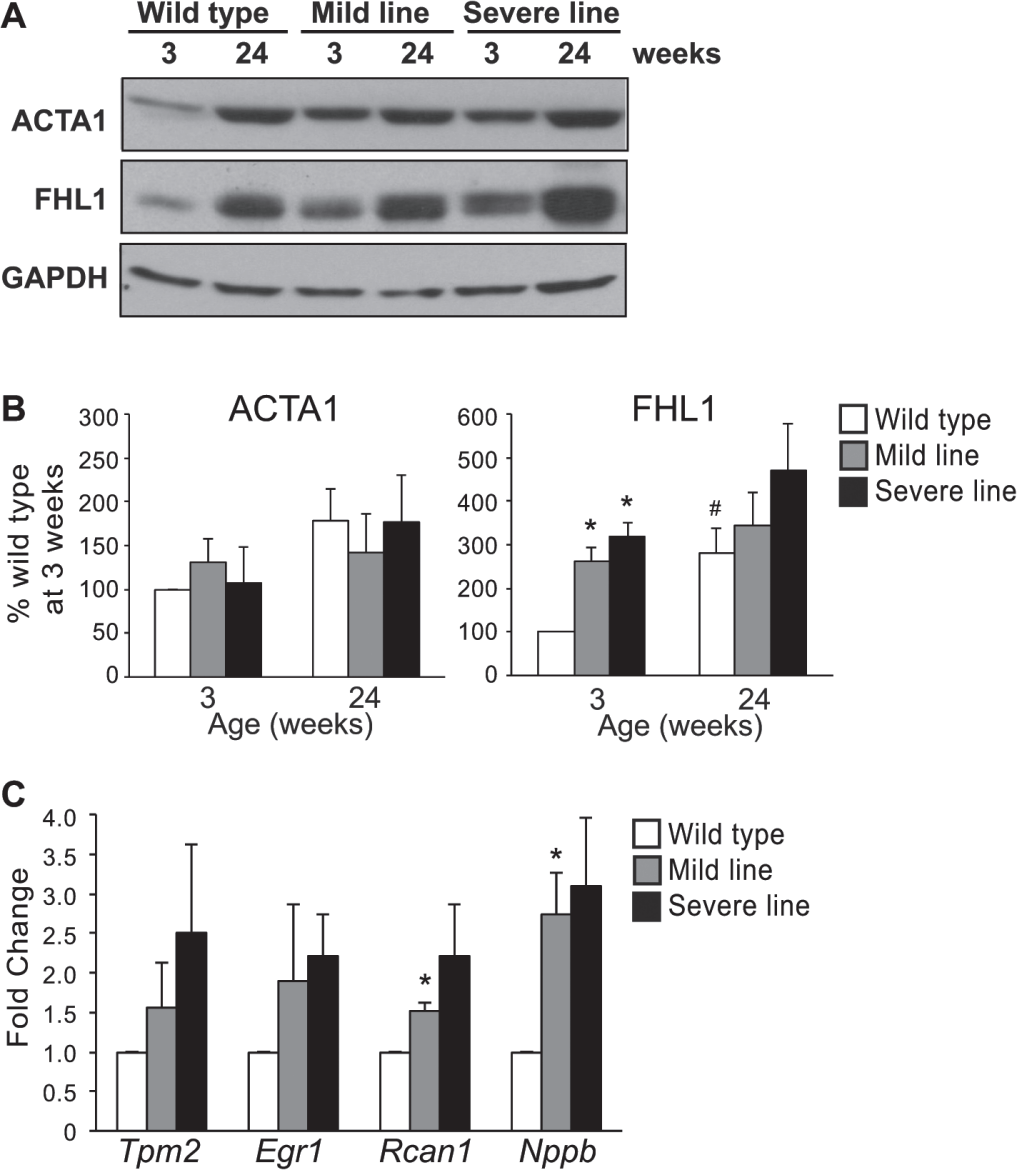
SRF target genes are less dysregulated at 24 than 3 weeks in the mild line. (A) Representative western blots of wild type, MHC-CELFΔ mild and severe line females at 3 and 24 weeks of age. Equivalent loading was further confirmed by Ponceau S staining (data not shown). (B) Quantitation of western blots (n = 3) shows an increase in the SRF targets ACTA1 and FHL1 in wild type hearts at 24 weeks compared to 3 weeks. Although ACTA1 and FHL1 levels are elevated in both transgenic lines at 3 weeks, levels are close to those observed in wild type at 24 weeks. An asterisk indicates a significant difference from wild type mice at the same age, and a pound sign indicates a significant difference from mice of the same group at 3 weeks (P ≤ 0.05). (C) Total RNA was extracted and mRNA levels were assayed by qRT-PCR using SYBR green-based detection. While *Tpm2*, *Egr1*, *Rcan1*, and *Nppb* levels were all significantly up-regulated in hearts of MHC-CELFΔ-574 mice at 3 weeks [8], only *Rcan1* and *Nppb* remain up-regulated in the mild line at 24 weeks. All four SRF targets showed trends towards increased expression in MHC-CELFΔ-10 mice at 24 weeks, but did not reach statistical significance. Fold changes shown represent the mean + standard error of the mean of three independent sample sets. An asterisk indicates a significant difference from wild type (P ≤ 0.05).

ACTA1 and FHL1 are two SRF targets that were previously shown to be up-regulated at the mRNA and protein levels in MHC-CELFΔ mice at 3 weeks [8]. ACTA1 and FHL1 protein levels increase with age in wild type mice (Fig 2A and 2B). FHL1 is elevated in the transgenic mice at 3 weeks, but at 24 weeks the levels in the mild line are comparable to wild type. FHL1 levels in the severe line show a strong trend towards still being elevated at 24 weeks, although this difference is not statistically significant (P = 0.10), perhaps due to greater variability in the levels in the severe line. ACTA1 levels exhibit a similar trend (i.e., elevated at 3 weeks and comparable at 24 weeks), but the differences are smaller, more variable, and do not reach statistical significance at either age. The levels of four additional SRF targets were evaluated at 24 weeks by qRT-PCR (Fig 2C). *Tpm2*, *Egr1*, *Rcan1*, and *Nppb* transcript levels are all significantly elevated in the mild line at 3 weeks, with *Egr1*, *Rcan1*, and *Nppb* all exhibiting greater than 3-fold higher levels than wild type [8]. At 24 weeks, although *Tpm2* and *Egr1* still show a trend towards slightly higher expression in the mild line, their levels are not significantly different from wild type (P > 0.2). *Rcan1* and *Nppb* remain significantly higher, although the extent to which *Rcan1* is up-regulated is approximately half that observed at 3 weeks. Together, these data indicate that there is a partial restoration of SRF target gene expression in the MHC-CELFΔ mild line at 24 weeks. In the severe line, all four SRF targets continued to show a trend towards increased expression compared to wild type at 24 weeks, but did not reach statistical significance (P = 0.15, 0.07, 0.10, and 0.07 for *Tpm2*, *Egr1*, *Rcan1*, and *Nppb*, respectively). The lack of statistical significance may be due to higher levels of variability in the severe line. Some degree of elevation was observed for all four transcripts in each sample of the three sample sets tested, ranging from 1.2- to 4.7-fold, 1.3- to 3.1-fold, 1.3- to 3.5-fold, and 1.4- to 4.0-fold for *Tpm2*, *Egr1*, *Rcan1*, and *Nppb*, respectively. Greater variability in the severe line was previously noted at 3 weeks as well [8].

It is important to note that while SRF activity may be partially restored in the mild line at 24 weeks, the fact remains that HOPX and FHL2 are more reduced in the mild line than the severe line in both young and mature mice. Several factors may contribute to the seeming discrepancy between the extent of SRF dysregulation and the severity of the cardiac phenotype, including differences in integration site of the transgene, genetic drift between the two lines, or even activation of a compensatory mechanism within the more affected severe line that is not triggered in the more functional mild line. We previously demonstrated, however, that the level of SRF activity did correlate with the development and severity of cardiomyopathy in different individuals within a line [8]. We also demonstrated that the degree of dysregulation of alternative splicing correlated with disease incidence and severity both within and between lines [8]. Thus, while SRF activity is likely to be a contributing factor, it is not sufficient to fully explain either the development of cardiomyopathy in MHC-CELFΔ mice or its reversal in the mild line.

### Global gene expression differences improve with age in the mild line

**Fig 3:**
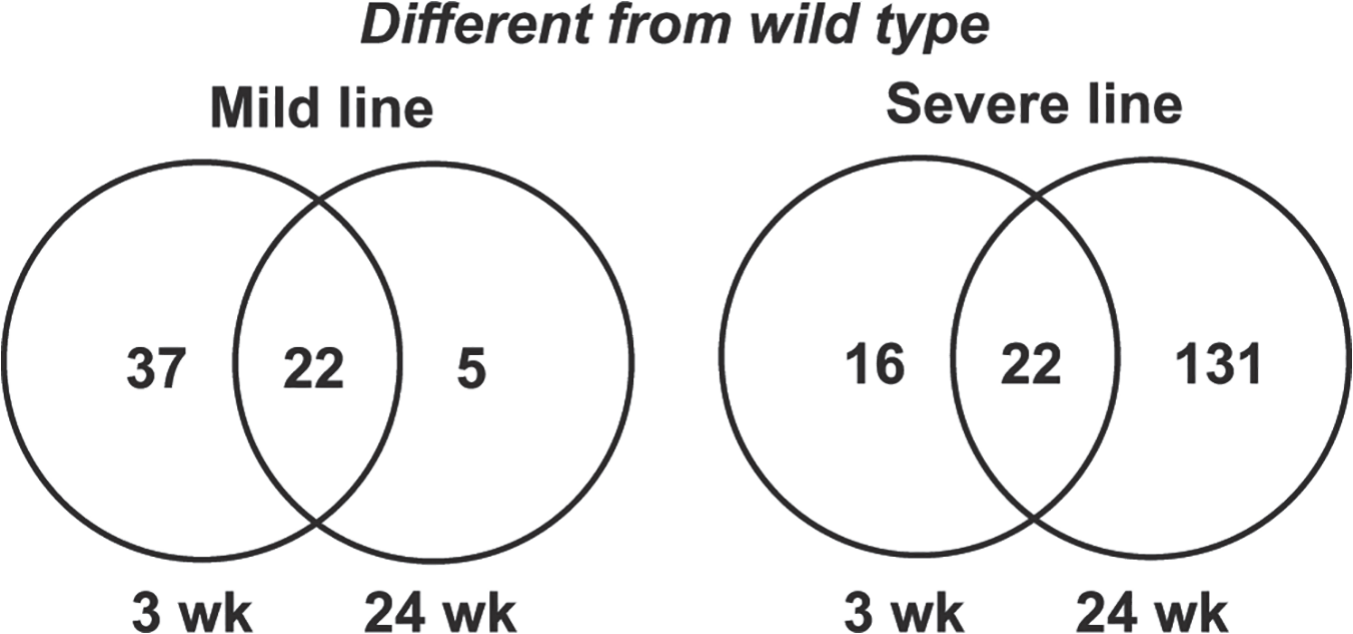
The number of affected genes in mild and severe lines at 3 and 24 weeks. Venn diagrams show the respective overlap of affected genes in MHC-CELFΔ-574 (“mild”) or MHC-CELFΔ-10 (“severe”) lines at 3 versus 24 weeks as compared to wild type mice at the same age.

To determine how global gene expression differences in MHC-CELFΔ mice change over time, we performed microarray analysis on total RNA extracted from wild type, MHC-CELFΔ mild and severe line hearts at 3 and 24 weeks of age. Wild type versus mild line and wild type versus severe line comparisons were made, and differentially expressed genes that exhibit an absolute fold change ≥ 2 were identified (S2 and S3 Tables, respectively). Differences at 3 weeks were then compared to differences at 24 weeks (Fig 3). Consistent with the reversal of cardiomyopathy, fewer genes differed from wild type in the mild line at 24 weeks than at 3 weeks. In fact, more genes returned to a normal level of expression than remained dysregulated. In the severe line where there is no functional recovery, however, global gene expression became more disrupted with age. For mild and severe lines, all of the genes that were dysregulated both at 3 weeks and 24 weeks showed changes in the same direction at the two ages, and most displayed a similar magnitude of change. This suggests that these sets of changes are not evoked as a consequence of either progressive degeneration (in the severe line) or compensation (in the mild line).

Notably, on the microarrays SRF targets that were significantly up-regulated in the mild line at 3 weeks were not up-regulated at 24 weeks, including not only *Egr1* and *Rcan1* (also known as *Dscr1*), but also *Ctgf* and *Fos* (S2 Table). In contrast, *Egr1* and *Ctgf* are more affected in the severe line at 24 weeks than at 3 weeks (S3 Table). This is consistent with at least partial normalization of the SRF program specifically in the mild line at 24 weeks, but the basis for this normalization is unclear. Although HOPX and FHL2 levels are higher in the mild line at 24 weeks than at 3 weeks (Fig 1C), they are still significantly lower than the wild type mice, and are lower than the more affected severe line. Thus, the expression of SRF targets cannot be explained solely by the level of inhibition of SRF by HOPX and FHL2. *Egr1* and *Fos* are immediate early response genes that are activated rapidly during induced heart failure and help mediate the cardiac stress response [20,21,22]. Although their induction is SRF-dependent, their expression can also be regulated in an SRF-independent manner via a microRNA, miR-499 [23]. Transgenic mice over-expressing miR-499 down-regulated the immediate early response genes *Egr1* and *Fos*, but up-regulated *Nppb* [23]. miR-499 lies within an intron of the *Myh7b* gene (also known as *Myh14*), which is up-regulated during surgically-induced cardiac hypertrophy in mice [24]. We did not detect differences in expression of the host *Myh7b* transcripts between wild type and the MHC-CELFΔ mild line by microarray or qRT-PCR (S2 Table and data not shown), but changes in expression of another microRNA might explain the restoration of some SRF targets, but not others, in these mice.

**Fig 4:**
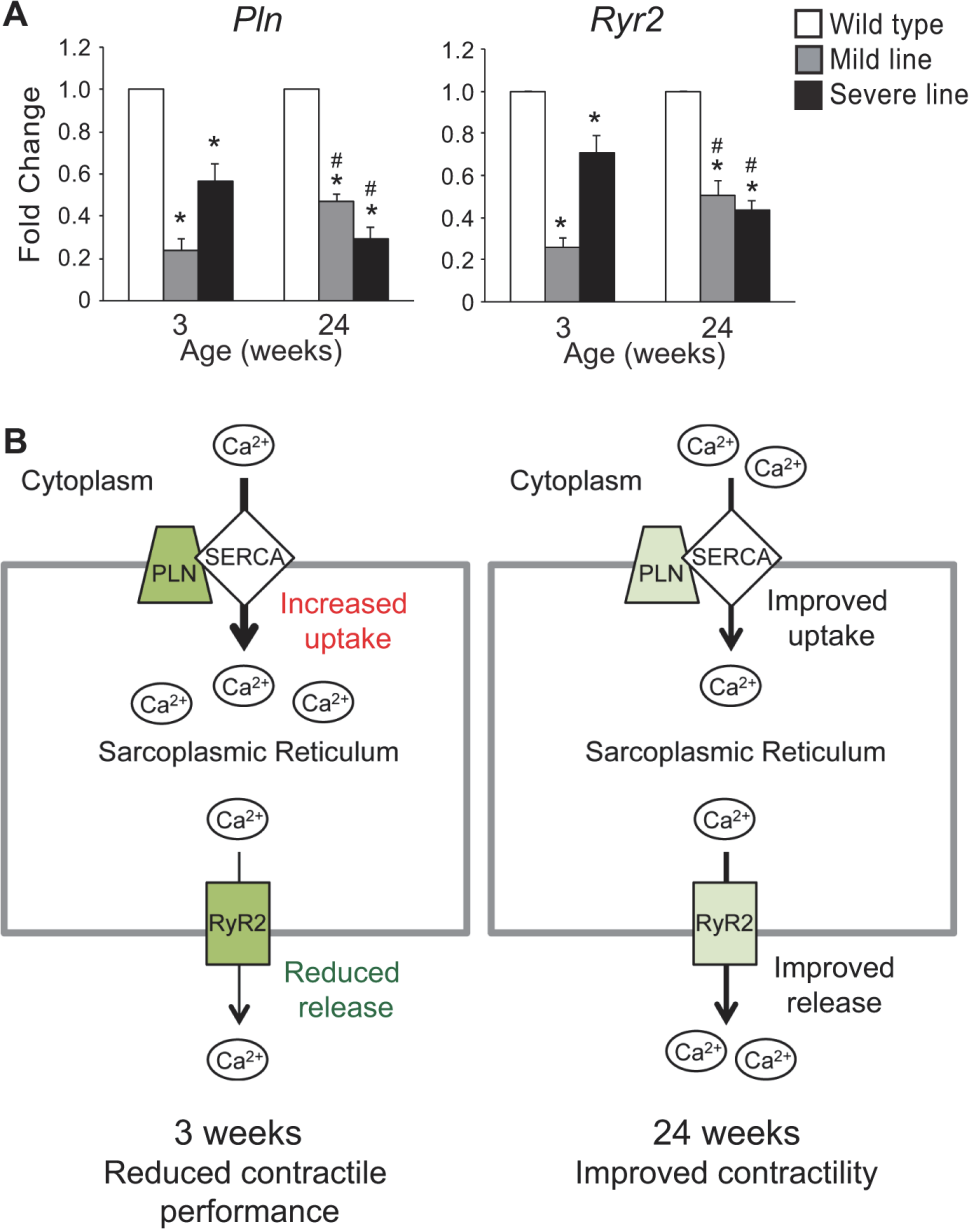
Calcium handling genes are less disrupted at 24 weeks than at 3 weeks in the mild line. (A) Total RNA was extracted and mRNA levels were assayed by qRT-PCR using SYBR green-based detection. *Pln* and *Ryr2* levels were reduced in both lines of transgenic mice compared to wild type, but while *Pln* and *Ryr2* levels went up in the mild line with age, they went down further in the severe line. Fold changes shown represent the mean + standard error of the mean of five independent samples for each group. An asterisk indicates a significant difference from wild type mice at the same age, and a pound sign indicates a significant difference from mice of the same group at 3 weeks (P ≤ 0.05). (B) In heart muscle, myofilament relaxation and contraction are driven by calcium cycling. Relaxation occurs when Ca^2+^ levels in the cytoplasm drop following uptake into the sarcoplasmic reticulum (SR), an intracellular calcium storage site, via SR or endoplasmic reticulum Ca^2+^-ATPase 2a (SERCA2a). Phospholamban (PLN) restricts Ca^2+^ uptake by inhibiting SERCA2a. An influx of calcium into the cell activates release of Ca^2+^ from the SR into the cytosol via the ryanodine receptor (RyR2), triggering contraction. Down-regulation (indicated by green coloring) of PLN and RyR2 in the mild line at 3 weeks would lead to a depletion of Ca^2+^ from the cytoplasm by increasing SERCA2a-mediated uptake and reducing RyR2-mediated release. This would result in reduced contractile performance. At 24 weeks, partial restoration of PLN and RyR2 to closer to normal levels would restore normal calcium handling and improve contractility.

In addition to these SRF targets, the reduction of other gene expression differences is also noteworthy. Gene ontology (GO) enrichment analyses previously identified genes involved in calcium handling and contractility as being affected in juvenile MHC-CELFΔ mice [8]. In particular, genes related to the sarcoplasmic reticulum (SR), the primary intracellular calcium storage site in striated muscle, were enriched in the mild line at 3 weeks [8]. In cardiac muscle, myofilament contraction and relaxation is driven by an ion-channel-mediated calcium cycle. Calcium is taken up into the SR by the SR or endoplasmic reticulum Ca^2+^-ATPase 2a (SERCA2a) pump [25]. Phospholamban (PLN) is a reversible inhibitor of SERCA2a activity, which is usually regulated through its phosphorylation status [25]. Release of Ca^2+^ from the SR occurs via activation of ryanodine receptor 2 (RyR2) by an initial influx of calcium into the cell through voltage-gated calcium channels at the plasma membrane [26]. RyR2-mediated Ca^2+^ release into the cytoplasm induces contraction; reuptake of Ca^2+^ into the SR by SERCA2a induces relaxation. Notably, the microarray results indicate that *Pln* and *Ryr2* expression are decreased to approximately half their normal levels in the mild line at 3 weeks, but are not significantly different from wild type at 24 weeks (S2 Table). To validate the microarray results, real-time RT-PCR was performed for *Pln* and *Ryr2* in wild type and MHC-CELFΔ hearts at 3 and 24 weeks (Fig 4A). At 3 weeks, *Pln* and *Ryr2* were both significantly reduced in the mild line. At 24 weeks, *Pln* and *Ryr2* levels were still reduced compared to wild type, but significantly less so than at 3 weeks. In the severe line, *Pln* and *Ryr2* levels were also reduced at 3 weeks, but to a lesser extent than in the mild line. In contrast to the mild line, however, the down-regulation of *Pln* and *Ryr2* in the severe line grew more rather than less pronounced with age (Fig 4A). Partial or complete ablation of PLN in mice leads to an increase in the uptake of Ca^2+^ by SERCA2a and changes in cardiac contractility [27,28,29]. Mutations that result in decreased *PLN* expression or loss of PLN function have been linked with dilated cardiomyopathy in humans [30,31,32]. RyR2 deficiency results in reduced intracellular Ca^2+^ levels, Ca^2+^ transient amplitude, and contractile function in mice [33,34]. Thus, the loss of *Pln* and *Ryr2* at 3 weeks would result in an increase in Ca^2+^ uptake into the SR and a decrease in Ca^2+^ release from the SR, respectively (Fig 4B). The resulting reduction in cytosolic calcium would impair contractility. Conversely, the partial recovery of *Pln* and *Ryr2* in the mild line at 24 weeks would presumably improve calcium cycling and contractility.

### Activation of compensatory gene expression changes are not observed in the mild line

**Fig 5:**
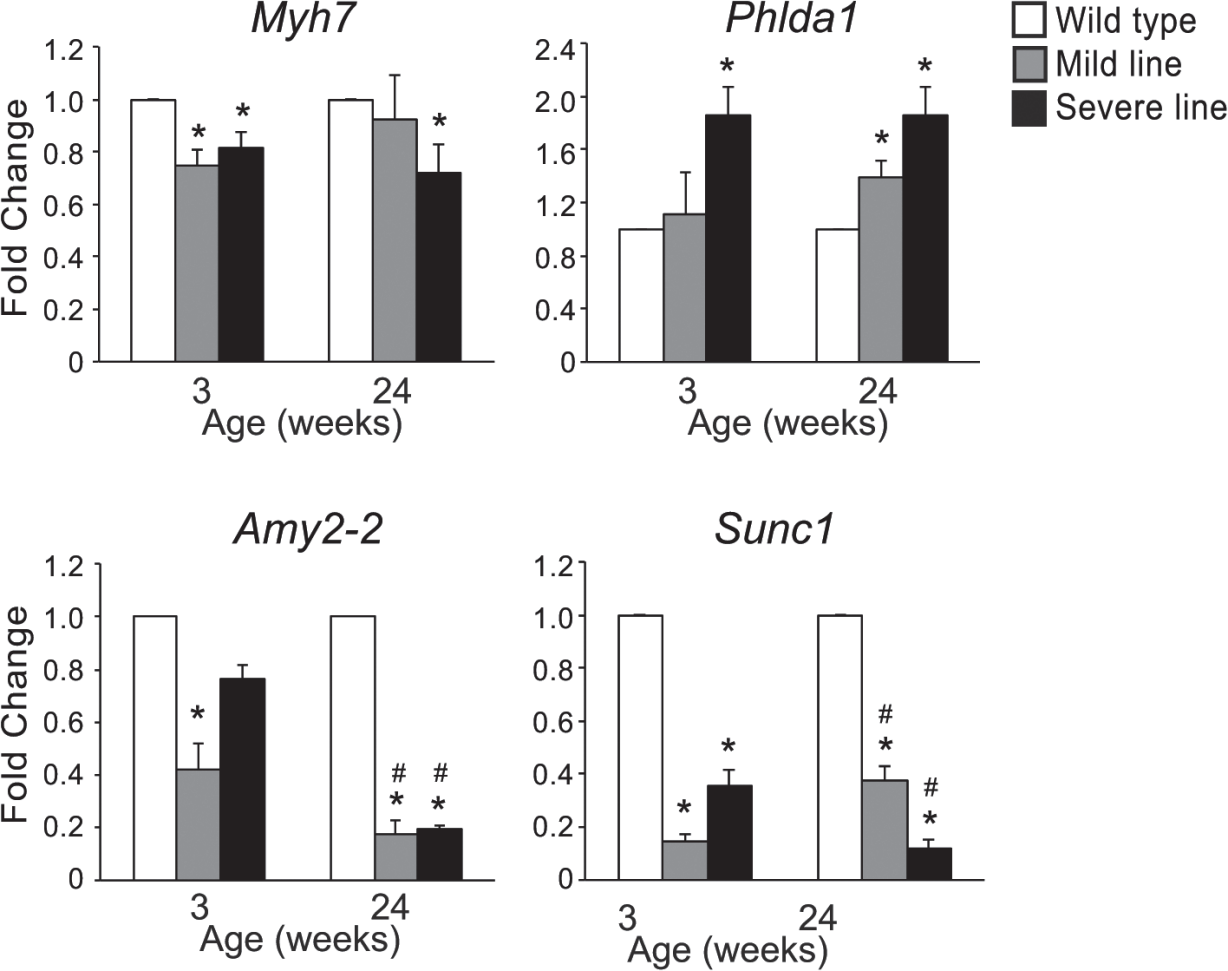
The expression levels of transcripts identified by microarray as affected at 24 weeks, but not 3 weeks, in the mild line were evaluated by real-time RT-PCR. Total RNA was extracted and mRNA levels were assayed using SYBR green-based detection. *Myh7* was mildly reduced in both mild and severe lines at 3 weeks, but was restored to normal levels specifically in the mild line at 24 weeks. *Phlda1* was specifically up-regulated in the mild line at 24 weeks, but was elevated in both juvenile and mature mice of the severe line. *Amy2-2* levels went down with age in both lines, whereas *Sunc1* levels improved in the mild line and worsened in the severe line with age. Fold changes shown represent the mean + standard error of the mean of four to five independent samples for each group. An asterisk indicates a significant difference from wild type mice at the same age, and a pound sign indicates a significant difference from mice of the same group at 3 weeks (P ≤ 0.05).

The partial restoration of normal gene expression in the mild line at 24 weeks is consistent with the improvement in cardiac function, but why this recovery occurs is unclear. One possibility would be that compensatory changes are induced at 24 weeks that are able to improve cardiac function and thereby reduce cardiac stress. From the microarray data, there were only five genes that were affected in the mild line at 24 weeks that were not affected at 3 weeks (S2 Table): *Myh7* and *Phlda1*, which were up-regulated, and *Amy2-2*, *Klk1b26*, and *Sunc1*, which were down-regulated. All five of these genes were similarly up- or down-regulated in the severe line at 24 weeks (S3 Table). To validate these changes, real-time RT-PCR was performed in wild type and MHC-CELFΔ hearts at 3 and 24 weeks (Fig 5). Although *Myh7* was not identified as being significantly different in MHC-CELFΔ hearts versus wild type at 3 weeks by microarray, small declines in *Myh7* expression were detected in both transgenic lines at this age. At 24 weeks, *Myh7* remained mildly reduced in the severe line. In the mild line, elevation of *Myh7* levels compared to wild type at 24 weeks indicated by microarray was not seen, although levels were no longer significantly lower than wild type. As seen by microarray, *Phlda1* levels in the mild line were indistinguishable from wild type at 3 weeks, but were significantly (although modestly) elevated at 24 weeks. *Phlda1* levels were higher in the severe line at both ages. Although not identified as affected in young mice by microarray, *Amy2-2* levels were significantly reduced in the mild line at 3 weeks, and showed a trend towards lower levels in the severe line. At 24 weeks, reductions in *Amy2-2* levels were significantly greater in both lines, consistent with the microarray results. *Sunc1* was reduced to a lesser extent in the mild line at 24 weeks compared to 3 weeks, whereas in the severe line down-regulation of *Sunc1* became more pronounced with age. We were unable to corroborate changes in *Klk1b26*, as *Klk1b26* transcripts were not detectable by real time RT-PCR in any of the samples using four different primer sets, even after increasing the amount of template (data not shown). Thus, we were able to confirm the up-regulation of *Phlda1* and down-regulation of *Amy2-2* in the mild line at 24 weeks. Although *Myh7* and *Sunc1* did not fully mirror the microarray results, they are in general agreement with the observation that gene expression changes lessen in the mild line with age. In and of itself, the fact that some of these transcripts are similarly up- or down-regulated in the severe line could indicate that they do not contribute to the recovery of the mild line, although it is also possible that the hearts of severe line mice may simply be too badly damaged at a young age to effectively recover regardless of what compensatory mechanisms may be activated. Let us therefore consider what is known in the literature about each of these genes in turn.

*Myh7* encodes the beta heavy chain of cardiac myosin. Changes in the relative abundance of alpha (fast) and beta (slow) myosin heavy chain affect the actomyosin kinetics of the myofibril, and a shift towards the beta isoform is thought to underlie reduced contraction velocity and relaxation in human heart failure [35]. Normalization of *Myh7* levels is associated with improved cardiac function and survival in rodent models of heart failure in which the microRNA miR-208a has been targeted [36,37]. Thus, while an up-regulation of *Myh7* suggested by the microarray results would not be consistent with the recovery of healthy function, the normalization of *Myh7* levels seen by real-time RT-PCR would be. *Phlda1* (also known as *Tdag51*) is a member of the pleckstrin homology-like domain family. *Phlda1* is induced by ER stress, and its over-expression promotes apoptosis [38]. It is not obvious how the increase in *Phlda1* would help recover cardiac function in the older transgenic mice.

There are also no obvious candidates for promoting recovery in the down-regulated genes. *Amy2-2* encodes amylase 2b, an enzyme that hydrolyzes alpha-glucoside bonds in polysaccharides, which can break down glycogen to produce glucose. Glucose is a preferred energy substrate in hypertrophic and failing hearts, and the increased utilization of glucose is thought to be beneficial for adaptive remodeling [39]. Loss of *Amy2-2* expression would therefore be predicted to reduce glucose availability and have a negative influence on the recovery of normal cardiac function. *Klk1b26* encodes a member of the kallikrein 1-related peptidase family of extracellular (chymo)tryptic-like serine proteases. KLK1b26 is involved in the maturation of epidermal growth factor (EGF) and renin [40]. EGF has been shown to be protective against ischemic injury in the heart [41,42]. Renin, on the other hand, is a protease involved in the production of angiotensin II, which induces systemic vasoconstriction, cardiomyocyte hypertrophy, and cardiac fibrosis, changes associated with worsening of ventricular function [43]. Thus, reduction in *Klk1b26* could have both negative and positive effects on recovery by reducing EGF and renin, respectively. Our failure to detect *Klk1b26* transcripts, however, suggests that it is expressed at very low levels in the heart. *Sunc1* encodes the sad1/unc-84 (SUN) domain-containing protein 1 (also known as SUN3). SUN domain-containing proteins are nuclear envelope proteins that physically connect the nucleus with the peripheral cytoskeleton and are involved in a variety of processes, including nuclear positioning and chromosome dynamics during meiotic cell division [44]. The specific functions of SUN3 have not been elucidated, but unlike other SUN domain-containing proteins SUN3 is distributed primarily in the endoplasmic reticulum and is expressed predominantly in the testes [45]. Thus, the consequences of changing *Sunc1* levels for cardiac function are unclear.

Although none of the changes in gene expression that occur specifically at 24 weeks seem overtly beneficial, we cannot rule out the idea that a compensatory pathway is activated that is able to offset the detrimental effects of misregulated splicing in the mild line. *Phlda1* or *Amy2-2* could have unappreciated effects on cardiac function. Or small changes in gene expression (i.e., less than the 2.0-fold cutoff used in the microarray analysis) could contribute to recovery. We also cannot rule out other kinds of compensatory changes that cannot be detected by microarray, such as alterations in miRNA expression, translational regulation, or post-translational modifications of proteins.

An alternative possibility to the activation of a compensatory pathway is that the aberrantly expressed splice variants could be more toxic in the cellular milieu of juvenile cardiomyocytes than that of adult cardiomyocytes [16]. In response to growth and changes in workload, the fetal versions of many proteins important for cardiac function are replaced by adult versions during early postnatal development [46,47]. For example, beta myosin heavy chain is predominantly expressed in the fetal heart, and is replaced by alpha myosin heavy chain in the adult heart [46]. The isoforms produced from inappropriate splicing could be functionally more compatible with the adult proteins than their fetal counterparts. Since there is no overt physical damage to the heart in the mild line (e.g., muscle death or fibrosis) [16], improved function due to enhanced compatibility might be sufficient to alleviate cardiac stress and restore a healthy status quo. We previously proposed that the creation of a new mouse model in which CELF activity is disrupted specifically during later adult life would distinguish between these recovery models. If repression of CELF activity in older mice results in a late onset dysfunction that then recovers, it would support a model in which an as-yet-unidentified compensatory pathway is activated. If, on the other hand, repression of CELF activity in older adults has no detrimental effect on cardiac function, this would support a model of stage-dependent incompatibility of aberrantly regulated splice variants. The creation and characterization of an inducible NLSCELFΔ-expressing transgenic mouse model remains a goal of future studies.

## Supporting Information

S1 Table Real time primer sequences used in this study. (PDF)

S2 Table Genes affected in the MHC-CELFΔ mild line (MHC-CELFΔ-574). (XLSX)

S3 Table Genes affected in the MHC-CELFΔ severe line (MHC-CELFΔ-10). (XLSX)
