# Supplementary material for: Gene Expression Analyses during Spontaneous Reversal of Cardiomyopathy in Mice with Repressed Nuclear CUG-BP, Elav-Like Family (CELF) Activity in Heart Muscle
Source: PLoS One. 2015 Apr 20;10(4):e0124462. doi: 10.1371/journal.pone.0124462 (PMC4404138; doi:10.1371/journal.pone.0124462)
Supplement: S1 Table — (PDF) [file pone.0124462.s001.pdf]

Table S1. Real time primer sequences used in this study

Genes	Forward primer	Reverse primer
Srf	cacctaccaggtgtcggaat	gtctggattgtggaggtggt
Hopx	caacttcaacaaggtcaacaagc	gcttaaaccatttctgcgtc
Fhl2	gccaggtacccgcaagatg	gcttctcatagcagggcacg
Tpm2	gagagcgagagaggaatgaagg	cctcagcgatgtgcttgg
Egr1	gagcgaacaaccctatgagc	tgggataactcgtctccacc
Rcan1	gctcagactttacacataggaag	gatcgtaatttatgacgggggtg
Nppb	gcacaagatagaccggatcg	cttcaaaggtggtcccagag
Pln	cccagctaagctcccataag	cagccaaatgtgagctgtctt
Ryr2	ccggtcttccactgacaaac	aggcaggatgtatggtccac
Myh7	catgctgaccttcctgcat	gcagaagaggcccgagtag
Phlda1	acctccaactctgcctgaag	gcaatgcactcttcccactt
Amy2-2	tggatttatgttggctcatcc	aggtggtccaatccagtcat
Sunc1	ctcaagtgcacccaaggagt	gaatggttgcttccattttg
Gapdh	tcgtcccgtagacaaaatgg	ttgaggtcaatgaaggggtc
